# Importance of medicine quality in achieving universal health coverage

**DOI:** 10.1371/journal.pone.0232966

**Published:** 2020-07-09

**Authors:** Sachiko Ozawa, Colleen R. Higgins, Tatenda T. Yemeke, Jude I. Nwokike, Lawrence Evans, Mustapha Hajjou, Victor S. Pribluda

**Affiliations:** 1 Division of Practice Advancement and Clinical Education, UNC Eshelman School of Pharmacy, University of North Carolina, Chapel Hill, NC, United States of America; 2 Department of Maternal and Child Health, UNC Gillings School of Global Public Health, University of North Carolina, Chapel Hill, NC, United States of America; 3 Promoting the Quality of Medicines (PQM) Program, United States Pharmacopeial Convention (USP), Rockville, MD, United States of America; Eberhard-Karls-Universitaet Tuebingen, GERMANY

## Abstract

**Objective:**

To assess the importance of ensuring medicine quality in order to achieve universal health coverage (UHC).

**Methods:**

We developed a systems map connecting medicines quality assurance systems with UHC goals to illustrate the ensuing impact of quality-assured medicines in the implementation of UHC. The association between UHC and medicine quality was further examined in the context of essential medicines in low- and middle-income countries (LMICs) by analyzing data on reported prevalence of substandard and falsified essential medicines and established indicators for UHC. Finally, we examined the health and economic savings of improving antimalarial quality in four countries in sub-Saharan Africa: the Democratic Republic of the Congo (DRC), Nigeria, Uganda, and Zambia.

**Findings:**

A systems perspective demonstrates how quality assurance of medicines supports dimensions of UHC. Across 63 LMICs, the reported prevalence of substandard and falsified essential medicines was found to be negatively associated with both an indicator for coverage of essential services (*p* = 0.05) and with an indicator for government effectiveness (*p* = 0.04). We estimated that investing in improving the quality of antimalarials by 10% would result in annual savings of $8.3 million in Zambia, $14 million in Uganda, $79 million in two DRC regions, and $598 million in Nigeria, and was more impactful compared to other potential investments we examined. Costs of substandard and falsified antimalarials per malaria case ranged from $7 to $86, while costs per death due to poor-quality antimalarials ranged from $14,000 to $72,000.

**Conclusion:**

Medicines quality assurance systems play a critical role in reaching UHC goals. By ensuring the quality of essential medicines, they help deliver effective treatments that lead to less illness and result in health care savings that can be reinvested towards UHC.

## Introduction

The goal of Universal Health Coverage (UHC) is to ensure that all people obtain the health services they need without suffering financial hardship when paying for them [1]. Sustainable Development Goal 3.8 supports UHC by aiming to achieve “access to safe, effective, quality, and affordable essential medicines and vaccines for all” [2]. A successful UHC system is a result of the combination of quality health services and expanding coverage of affordable care.

Quality health services cannot be delivered without quality-assured medicines. Ensuring medicine quality is paramount in providing safe and effective health care and reducing overall health care costs. The World Health Organization (WHO) estimates that, on average, 1 in 10 medical products circulating in low- and middle-income countries (LMICs) is substandard or falsified [3]. WHO defines substandard medicines as “authorized medical products that fail to meet their quality standards, specifications, or both [4].” Medical products that “deliberately and fraudulently misrepresent their identity, composition, or source” are classified as falsified [4]. A recent meta-analysis found that 13.6% (95% CI, 11.0–16.3%) of essential medicines in LMICs were either substandard or falsified [5], with other literature reviews reporting a comparable range [3, 6–8]. Large percentages of poor-quality antimalarials (19.1%) and antibiotics (12.4%) were found, with the highest reported prevalence observed in Africa (18.7%) and Asia (13.7%) [5]. Despite the growing evidence of the problem, the importance of quality-assured medicines and the challenge of ensuring their quality is rarely discussed in UHC planning.

The pharmaceutical system operates within a complex health system, where pharmaceutical good governance can be viewed as a component of health systems strengthening necessary to support UHC [9]. Ensuring the delivery of quality-assured medicines also requires strengthened governance of medicine procurement systems. Medicine procurement for the public sector is typically handled by the government, where purchase and supply chain delivery are shared between medicines and other health service commodities. Medicines are a leading source of health system inefficiency due to the pervasiveness of inappropriate use, variable quality on the market, and high-priced brand name medicines being preferred over generics despite their bioequivalence [9]. In addition, availability of unregistered medicines presents alternatives for patients to access and use medicines that are neither included in health systems nor covered on insurance plans. Improving financing schemes and strengthening governance of medicine procurement systems is essential to increasing financial protection and access to health services, which are core tenets of UHC [10]. Increased health care utilization alone will not result in better outcomes if the quality of services is low [11]. Similarly, the full benefits of expanded coverage may not be realized without also ensuring the quality of covered medicines [12].

Financing and procurement of medicines play an important role in UHC schemes, where medicine quality needs to be safeguarded [9]. Globally, a quarter of all health expenditures are spent on medicines [13]. *The Lancet*’s Commission on Essential Medicines Policies estimated that between $13 and $25 per capita (US$77.4 to $151.9 billion) is required to finance a basic package of essential medicines in all LMICs [13]. However, the majority of low-income countries and over a quarter of middle-income countries spend less [13]. Moreover, medicines are often paid out-of-pocket in many countries, putting individuals and households at risk of having poor access to treatments and/or becoming poor due to their costs [14–16]. UHC can improve population health by providing quality health services and quality-assured medicines, while preventing catastrophic medical expenditures for the world’s poorest communities.

This study uses systems mapping to conceptually illustrate the benefits of ensuring medicine quality in UHC. We subsequently examined the association between UHC and medicine quality in the context of essential medicines in LMICs using data on substandard and falsified medicines prevalence and UHC indicators. The study also demonstrates the health and economic costs of poor-quality medicines that could be averted by quality assurance interventions where savings could be reinvested in UHC, using antimalarials as a case study.

## Materials and methods

### Systems map linking medicine quality with UHC

Systems mapping has been increasingly used in health to help understand indirect effects in complex systems [17–20]. With more detail displaying associations between variables than in a conceptual framework, systems mapping can show how different parts of a system fit together and interact, which make it a useful tool for understanding linkages that are less frequently explored. An advantage of a systems approach, such as systems mapping, is that it takes the entire system into consideration, which can facilitate understanding of indirect effects and unintended consequences [17]. Taking a systems perspective is particularly useful for medicine quality due to the variety of processes and stakeholders involved, from medicine manufacturing, to purchase and utilization throughout the supply chain. As medicine quality is not commonly emphasized in UHC planning and policies, we use a systems approach to make a conceptual linkage between medicine quality assurance and UHC, allowing us to explore the potential secondary and tertiary effects of medicine quality assurance systems and interventions on UHC.

This research mapped the series of stages where quality assurance processes and interventions are necessary to ensure the quality of medicines reaching patients, illustrating the flow of medicines from manufacturing to utilization. We illustrated the linkages between health systems and health insurance processes, and the resulting benefits to patients. The systems map also illustrates the three dimensions of UHC, demonstrating how medicine quality assurance processes and interventions, and the ensuing benefits, relate to the UHC dimensions [10]. Finally, we depicted interventions that target quality assurance processes to illustrate some of the key levers required to ensure quality-assured products in UHC. We developed the systems map through an iterative process. First, the study team mapped the system map elements based on existing literature. We then solicited feedback on the map from external stakeholders with expertise in health systems and medicine quality assurance, including experts from the Promoting the Quality of Medicines (PQM) program at the United States Pharmacopeial Convention (USP), United States Agency for International Development (USAID), and academics at the University of North Carolina at Chapel Hill (UNC). We subsequently revised the systems map based on conceptual feedback by including additional content and linkages between the map elements, and made structural revisions to improve comprehension and readability of the map.

### Association between UHC and medicine quality

To relate the conceptual linkage between UHC and medicine quality portrayed through our systems map to real world indicators, we investigated the potential association between UHC and medicine quality indicators utilizing existing data. Two indicators from the WHO were used to determine progress towards UHC: an indicator on essential services coverage; and an indicator on the proportion of the population with large household expenditures on health as a share of total household expenditures [21]. The WHO indicator on coverage of essential services was calculated from 16 tracer indicators measuring average insurance coverage of interventions in areas such as maternal and child health, infectious diseases, and non-communicable diseases [22]. The WHO indicator for large health expenditures measured the proportion of the population that spends over 10% of their household expenditures on health services.

In addition, two indicators for government effectiveness and regulatory quality were abstracted from the World Governance Indicators of the World Bank [23]. The government effectiveness measure combined perceptions of the quality of a country’s public and civil services, independence from political pressures, as well as formulation and commitment to policies. This indicator is also used by USAID to help inform strategic decisions and assess a country’s path to self-reliance [24]. The second regulatory quality indicator from the World Bank assessed the perception of the soundness of a government’s policies and control over private sector practices. We also retrieved under-five mortality rates for each country from the United Nations International Children's Emergency Fund (UNICEF) [25].

We examined how these indicators were associated with estimated prevalence of poor-quality medicines for each country, using data on reported prevalence of substandard and falsified essential medicines among 63 LMICs previously gathered from a systematic literature review and meta-analysis [5]. Substandard and falsified medicines prevalence was defined as the number of failed samples over the total number of samples of essential medicines chemically tested and publicly reported within each country (S1 Table). We examined the association between country specific reported prevalence of substandard and falsified medicines and the proportion of essential services covered, government effectiveness, regulatory quality, large health expenditures, and under-five mortality. We used simple linear regression models given the small sample size for a multi-country analysis, using prevalence of substandard and falsified medicines as the dependent variable. We conducted visual tests for linearity and Breusch Pagan tests for heteroskadasticity. Gross Domestic Product (GDP) per capita was assessed as a main confounder in each analysis.

### Health and economic impact of poor-quality antimalarials

To illustrate the potential health and economic impact that can result from ensuring the quality of antimalarials, we analyzed the current landscape of the impact of poor-quality antimalarials in a case study. Four countries were included in our analysis: the Democratic Republic of the Congo (DRC), Nigeria, Uganda, and Zambia [26–28]. Malaria is associated with high levels of morbidity and mortality in LMICs, and medicines to treat malaria (i.e. antimalarials) are, within the therapies surveyed, one of the medications most commonly tested and found to be of poor quality [5]. UHC planning in malaria endemic countries would naturally entail decisions on what malaria medications and services to make available, and what populations will receive these benefits.

Estimates of country-specific impact were obtained from the Substandard and Falsified Antimalarial Research Impact (SAFARI) model, an agent-based model we built that simulates malaria disease progression, care seeking, and outcomes for children under age five [26–28]. The SAFARI model was adapted and run separately for each country using country-specific demographic and epidemiological inputs. Methods for the development of the SAFARI model and individual country data are described in detail in other publications [26–28]. We simulated child agents who sought malaria treatment and received either quality-assured or poor-quality antimalarials. Poor-quality antimalarials reduced treatment efficacy and increased the likelihood of agents progressing to severe malaria. The model tracked agent children’s health over the course of treatments leading to hospitalization, neurological sequelae, death, further treatment, or recovery (S1 Fig). The costs incurred from these events were recorded as both direct costs to patients and facilities for the treatment of malaria, as well as the indirect productivity losses incurred from time spent seeking treatment, life lived with a neurological disorder caused by severe malaria, and early death due to malaria.

In order to compare the impact of poor-quality antimalarials across countries, we calculated the potential savings in each country if the reported prevalence of substandard and falsified antimalarials were to be reduced by 10%. We estimated this impact by comparing a baseline scenario using the reported prevalence of substandard and falsified antimalarials, to a scenario where 10% more antimalarials were quality-assured. This provided an estimate of the health and economic benefits a health system would experience by investing in quality assurance mechanisms that increased the supply and utilization of quality-assured antimalarials and reduced use of substandard and falsified antimalarials by 10%. The savings are estimated in fewer deaths, fewer hospitalizations, lower costs of care, and productivity gains simulated when poor-quality antimalarials are replaced with quality-assured antimalarials. The economic impact of poor-quality antimalarials was further assessed at an individual level to demonstrate the cost of poor-quality medicines per malaria case, and the cost attributable to substandard and falsified medicines for each additional malaria death, hospitalization, and disability-adjusted life year (DALY). This was calculated by taking the total savings in direct costs and productivity losses averted from reducing the prevalence of substandard and falsified antimalarials by 10% and dividing it by estimated reductions in deaths, hospitalizations, and DALYs.

To contextualize the impact of improving medicine quality, we compared a scenario in which no poor-quality antimalarials were present, to other options that governments may consider in reducing malaria burden. We simulated two additional interventions: a scenario in which there were no stockouts of antimalarials, and one in which only Artemisinin-based Combination Therapies (ACTs) were taken as first-line therapy. Moreover, low-quality anti-infectives can induce further costs to health systems and society by contributing to the development of antimicrobial resistance. We simulated the impact of widespread ACT resistance in a hypothetical scenario where the efficacy of ACTs were reduced to efficacy of other malaria treatments, impacting the duration and severity of malarial illness among children.

## Results

### Systems map linking medicine quality with UHC

**Fig 1:**
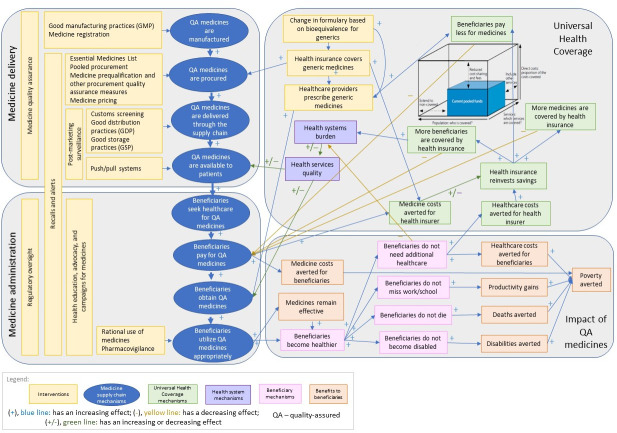
Systems map linking medicines quality with UHC.

Fig 1 presents the systems map of associations between medicine quality and essential components of UHC. We first mapped the essential steps and processes involved in order for beneficiaries to receive quality-assured medicines (blue ovals). This illustrates the movement of medicines from manufacturing, procurement, and through the supply chain to reach health facilities, after which beneficiaries who seek health care can obtain and utilize medicines. We then mapped the resulting benefits–when beneficiaries utilize quality-assured medicines, as opposed to no medicines or poor-quality medicines, beneficiaries can be healthier with a shorter duration of illness and milder symptoms, thus needing less additional health care and having the possibility to return to work earlier (pink rectangles) [26–29]. For example, using a substandard medicine with inadequate amounts of active pharmaceutical ingredients could add days to recovery time or be completely ineffective, requiring a patient to seek additional care or suffer a longer duration of illness without proper treatment, increasing the severity of disease [26–28]. With quality-assured medicines, beneficiaries may be cured of illness, and may even avert disability or death [26–28].

Quality-assured medicines bring further benefits (orange rectangles). Appropriate use of quality-assured medicines contributes to maintaining medicine efficacy by delaying the development of antimicrobial resistance [29, 30]. When beneficiaries require less health care because they have access and utilize quality-assured medicines, they decrease the risk of becoming poor due to additional expenses for medicines and health care [31]. Healthier beneficiaries are also more productive in society, resulting in productivity gains. Ensuring medicine quality thus contributes to the overall goal of UHC to ensure health care access without suffering financial hardship when paying for them.

Averting the need for additional health care when beneficiaries are healthier with quality-assured medicines can trigger a number of health system mechanisms (purple rectangles). When beneficiaries require less health care, the health system is less burdened and the quality of health services could improve [32]. Healthier beneficiaries utilizing quality-assured medicines can also trigger UHC mechanisms (green rectangles). As beneficiaries require less care, health care costs for health insurers decrease, and those savings could be reinvested back into the system [33]. Health insurers can reinvest these savings into three dimensions of UHC–as illustrated by the cube in Fig 1 –by covering more medicines and services, covering more beneficiaries, or covering more costs incurred by individuals to reduce cost-sharing for medicines and health services.

Regulatory oversight and quality assurance mechanisms throughout the supply chain reinforce the system that ensures patients can obtain and use quality-assured medicines. These are the backbone of the key interventions (yellow rectangles) within the system, broken down to be those affecting medicine delivery, administration, or UHC. These interventions include best practices (e.g. good manufacturing, distribution, and storage practices), policies (e.g. for medicine registration, pre-qualification requirements, and procurement), regulations (e.g. post-market surveillance, including customs screening and inspections), and education (e.g. health education on medicines and advocacy campaigns) [34–36]. Within UHC, we include the role of formulary management, where bioequivalence studies can trigger changes in formularies [37, 38]. This can subsequently change insurance coverage for generic medicines, affecting prescribing practices and costs to health insurers and beneficiaries [39–42]. Getting quality-assured generic medicines on the preferred list of formularies is an example of a medicine quality assurance intervention that can save costs to health insurers and affect UHC [41, 42].

The systems map connects the mechanisms to build effective medicines quality assurance systems with the health and economic benefits that quality-assured medicines can bring to UHC, highlighting the need for investments in strengthening these systems to achieve UHC.

### Association between UHC and medicine quality

We sought evidence to further describe the linkages between medicine quality and UHC by examining the association between existing data on the reported prevalence of substandard and falsified medicines (S1 Table) and UHC indicators among 63 LMICs (Table 1). Each individual indicator was first regressed on prevalence of substandard and falsified medicines while controlling for GDP per capita. This confounder was only found to yield a strong relationship when included with the indicator for large health expenditures. Visual tests for linearity were conducted and yielded no grounds to reject the linearity assumption. Breusch Pagan tests for heteroskadasticity were performed on each model, each resulting in insignificant p-values (>0.05). With no evidence to reject homoskedasticity, we present the results of simple linear regressions.

**Table 1:** Summary of linear models of associations of UHC indicators with reported prevalence of substandard and falsified essential medicines.

Variable^1^	Coefficient	Standard error	*p* value
Proportion of essential services covered^2^ N = 63	-0.002	0.001	0.054
Proportion of households with health expenditures of 10% or more of household expenditure^1^	-0.002	0.002	0.335
GDP Per CapitaN = 51	2.081e-05	5.210e-06	<0.001
Indicator for Government Effectiveness^3^ N = 63	-0.061	0.030	0.048
Indicator for Regulatory Quality^3^ N = 63	-0.076	0.037	0.046
Under-Five Mortality Rate N = 63	0.001	0.001	0.056

GDP: gross domestic product

^1^ Data from the most recent year available were used for each country for each indicator: coverage of essential services: 2015; large household expenditure: various years between 1998 and 2015; government effectiveness: 2017; regulatory quality: 2017; under-five mortality rate: 2017.

^2^ Data were retrieved from the Global Health Observatory repository

^3^ Measured on a linear scale between -2.5 and 2.5

**Fig 2:**
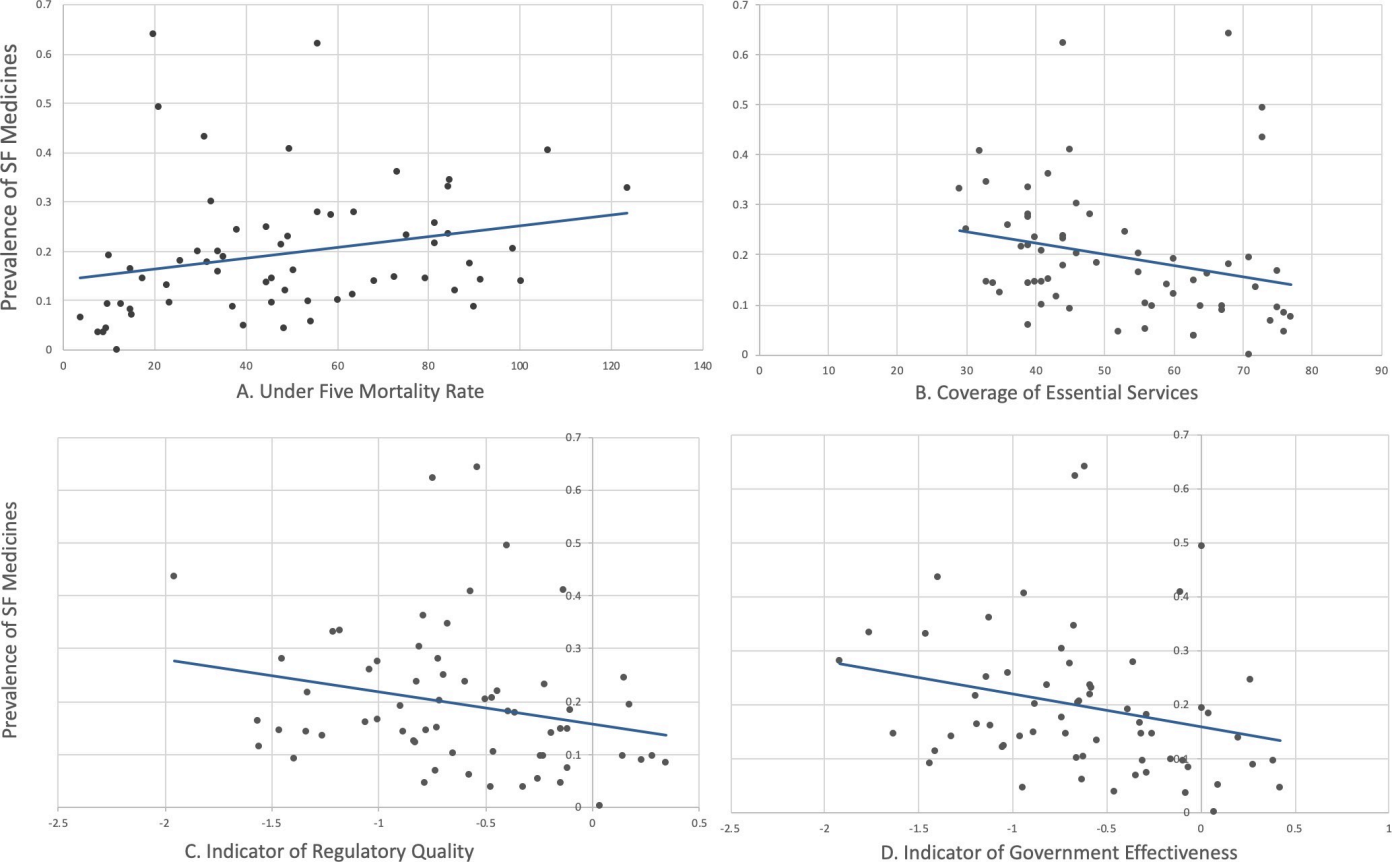
Association of reported country prevalence of substandard and falsified medicines with UHC indicators. Regulatory quality (C) and government effectiveness (D) are measured on a linear scale between -2.5 and 2.5.

Across countries, we found that coverage of essential services by health insurance schemes was found to be negatively associated with reported prevalence of poor-quality medicines (*p* value 0.054; Fig 2B). The direction of the relationship could indicate that countries that have higher coverage of essential services by health insurance were likely to have lower reported prevalence of poor-quality medicines. However, the effect size was small and not significant at a 0.05 alpha level. Countries with stronger capacity to provide greater health insurance coverage could be in a better position to effectively regulate the medicines supply chain, thus preventing the availability of poor-quality medicines. Coverage of essential services has a further benefit when combined with medicines quality through streamlined, well-regulated processes. This mitigates the need to seek potentially compromised medicines from the informal system.

A negative association was also found between reported prevalence of substandard and falsified medicines and indicators for government effectiveness and regulatory quality, where higher reported prevalence of poor-quality medicines was associated with lower governmental effectiveness (*p* value 0.048; Fig 2C) and lower regulatory quality (*p* value 0.046; Fig 2D). The direction of this association reflects the expected inverse relationship between these parameters, where an increase in regulatory quality or government effectiveness scores was associated with a decrease in prevalence of poor-quality medicines, though with a small effect size. In addition, reported prevalence of substandard and falsified medicines was shown to have a slightly positive but not significant correlation with under-five mortality (*p* value 0.056; Fig 2A).

No significant association was found between large health expenditures and reported prevalence of substandard and falsified medicines (*p* value 0.335) after controlling for GDP per capita. This lack of observable association does not indicate that poor-quality medicines do not result in larger household expenditures. It instead suggests that, given the data available, poor-quality medicines may not be the main driver of enhanced expenditures where other factors are at play. Furthermore, data on catastrophic health spending was not available for all countries, resulting in a lower number of observations for this analysis compared to others (51 compared to 63 countries).

### Health and economic impact of poor-quality antimalarials

Table 2 summarizes the results of the SAFARI model, showing the monetary and health savings that could result if the prevalence of poor-quality antimalarials in each modeled country were reduced by 10%. The reported prevalence of substandard and falsified antimalarials in the four countries ranged from 10.3% in Zambia to 22% in Uganda [5]. By improving the quality of antimalarials through removing 1 in 10 poor-quality ones, we simulated fewer deaths annually– 8,255 averted in Nigeria, 507 in Uganda, 208 in Zambia, and 667 and 4,764 in the Kinshasa and Katanga regions in DRC. This means that ensuring that 10% more antimalarials are quality-assured can result in 22 fewer deaths per day in Nigeria and 3 fewer deaths a week in Zambia.

**Table 2:** SAFARI model results for the annual impact of substandard and falsified antimalarials in four countries.

			Impact of 10% Reduction in reported SF Prevalence									
Country	GDP per capita (2017 USD)	Reported Prevalence of SF Antimalarials	Fewer Deaths^1^	Fewer Hospitalizations	Fewer DALYs	Savings in Direct Costs^2^	Savings in Productivity	Total Cost Savings	Cost of SF per Case of Malaria^4^	Cost per Death due to SF	Cost per Hospitalization due to SF	Cost per DALY due to SF
Nigeria	$1,968	14.9%	-8255	-22,349	213,784	-$20,030,671	-$578,450,134	-$598,480,805	$37	$72,499	$26,779	$2,799
Katanga (DRC)^3^	$463	19.1%	-4764	-66,806	-	-$9,999,476	-$58,115,183	-$68,114,659	$86	$14,286	$1,019	-
Kinshasa (DRC)^3^	$463	19.1%	-667	-18,743	-	-$2,601,414	-$8,366,492	-$10,967,906	$51	$16,413	$584	-
Uganda	$607	22.1%	-507	-6,298	48,273	-$2,330,741	-$11,700,689	-$14,031,430	$9	$27,668	$2,228	$291
Zambia	$1,513	10.3%	-208	-914	5,649	-$813,392	-$7,514,948	-$8,328,340	$7	$40,103	$9,116	$1,474

GDP: Gross domestic product, DRC: Democratic Republic of the Congo, SF: substandard and falsified, DALYs: disability adjusted life years

^1^ Table results were calculated by comparing baseline results for the health and economic burden of malaria to a scenario in which only high quality antimalarials were available.

^2^ Costs are presented in 2017 USD. Lifetime costs were discounted at 3%.

^3^DALY estimates were not included in the DRC version of the SAFARI model.

^4^Estimated by dividing the costs of substandard and falsified antimalarials by the number of malaria cases in each country.

We estimated that each country would experience substantial savings by improving the quality of antimalarials for children. By replacing 10% of the current substandard and falsified antimalarials with quality-assured antimalarials, we simulated savings of $8.3 million in Zambia, $14 million in Uganda, $598 million in Nigeria, $68 million in Katanga and $11 million in Kinshasa. Direct costs incurred by public facilities and by patients were estimated to contribute between 3.3% (Nigeria) and 23.8% (Kinshasa) of total savings ($813 thousand to $20 million) across countries. The model results suggest that assuring the quality of antimalarials would result in considerable savings in productivity, largely composed of the potential economic productivity that a child would contribute over a lifetime if death or disability due to poor-quality antimalarials were averted. We estimated that reducing poor-quality antimalarials by 10% would reduce productivity losses annually by $7.5 million in Zambia and $578 million in Nigeria.

We estimated that substandard and falsified antimalarials contribute between $7 per malaria case in Zambia, and $86 per malaria case in the Katanga region in DRC. Each additional death due to poor quality antimalarials approximately cost between $14,300 per death in Katanga, and $72,500 per death in Nigeria. Each additional pediatric malaria hospitalization attributable to substandard and falsified medicines cost between $584 per hospitalization in Kinshasa, and $26,800 per hospitalization in Nigeria. The costs at an individual level are heavily influenced by the cost of care, amount of care seeking, number of malaria cases, and the GDP per capita in each country.

**Fig 3:**
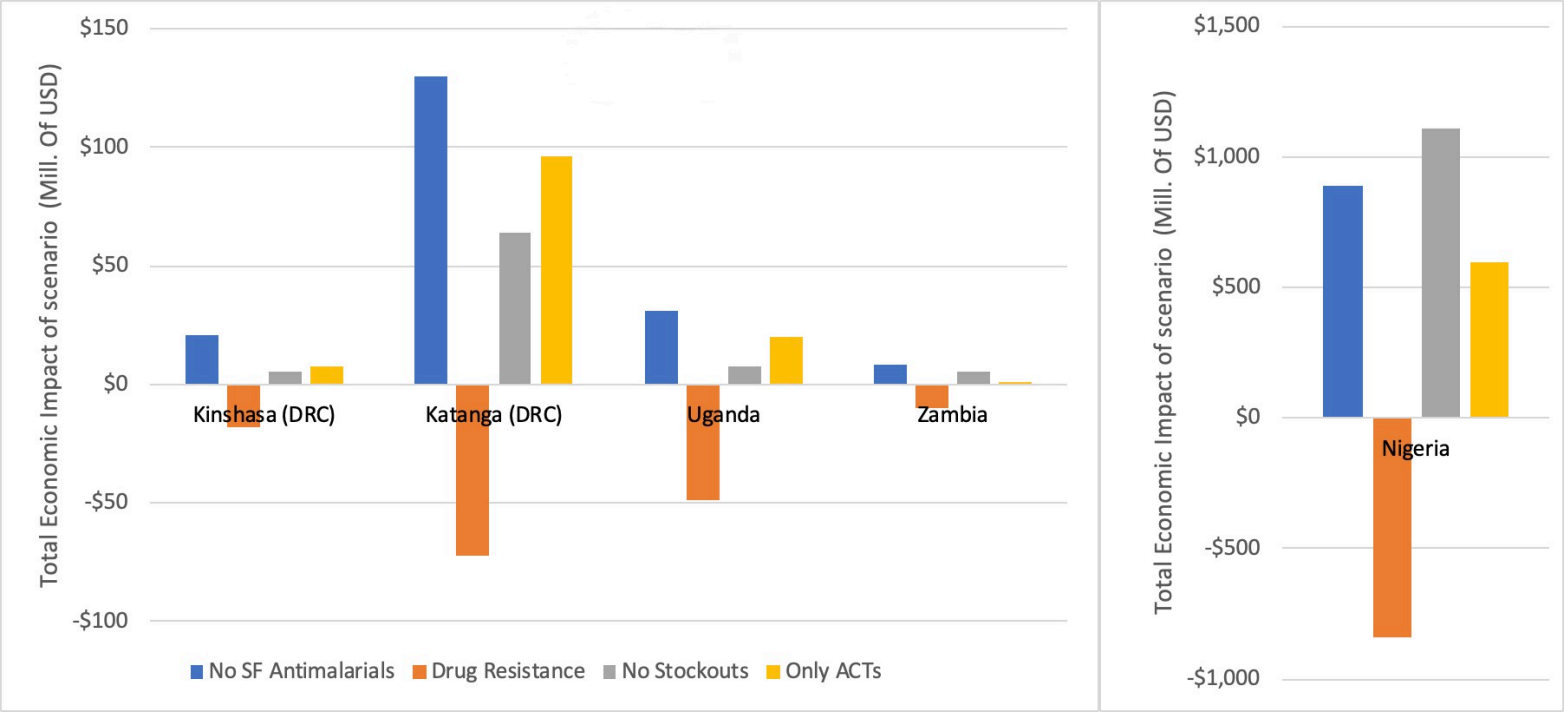
Total economic impact of simulated scenarios. Nigeria is depicted separately due to scale. Costs are in 2017 USD.

Fig 3 compares the potential economic impact of three simulated interventions (no substandard and falsified antimalarials, no stockouts, and replacing all antimalarials with ACT treatments) and one negative scenario (emergence of antimalarial resistance) in each country. Among the different investments that governments could make towards malaria burden reduction, ensuring the quality of all antimalarials was found to be the most impactful course in DRC, Uganda, and Zambia, while in Nigeria it was second to preventing stockouts. In addition, the negative impact of antimalarial resistance, a potential consequence of recurrent use of poor-quality medicines, could have a substantial negative impact, costing countries nearly $10 million in Zambia to $839 million in Nigeria.

## Discussion

Despite the many differences between countries, this study consistently observed that substandard and falsified essential medicines were associated with key components of UHC such as coverage of essential medicines, government effectiveness, and regulatory quality. Using systems mapping, we illustrated the conceptual link between UHC and regulatory measures to improve the quality of medicines. With the conceptual mapping as guidance, we demonstrated that a relationship can be observed between medicine quality and UHC indicators within existing data. We then showed the importance to a health system of investing in the quality of medicines, and the potential savings that could be reinvested in UHC dimensions (i.e. covering more beneficiaries in health insurance schemes, covering more services, or reducing out-of-pocket expenditures for patients). We show that improving antimalarial quality would not only save millions by improving outcomes—it can also help avert the immense costs associated with potential development of drug resistance [43]. Using high-quality medicines results in shorter and less severe illness, leading to fewer fatalities and less opportunity for the spread of contagious diseases. With less time spent sick, and less money spent on care resulting from poor-quality medicines, patients can be more economically productive and less likely to use up their resources to pay for health care. Moreover, improving medicine quality can have a dual impact by reducing inequities, since providing only high-quality antimalarials has been shown to have greater benefits among poor and rural populations [44].

Interventions aimed at ensuring medicine quality, such as good practices in manufacturing and distribution, or establishing an essential medicines list, strengthen institutions necessary for maintaining continued access to quality-assured medicines and quality services [45–48]. UHC financing schemes can create incentives for providers to prescribe and dispense medicines procured through systems or processes that ensure their quality. As coverage of services and populations increases, this system may require continuous strengthening to satisfy the increased demand for quality-assured medicines. Some quality assurance and regulatory interventions for medicines could also result in cost savings to health insurers, facilitating greater coverage of essential services. For example, data from bioequivalence studies of quality-assured generic medicines may guide and facilitate registration and subsequent procurement of cheaper generics [37, 38], resulting in cost savings.

Recent literature has drawn attention to potential negative unintended consequences in UHC implementation, where incentives for procuring cheaper medicines to meet increased demand under UHC can lead to arbitrage opportunities and proliferation of suppliers of cheaper, non-quality-assured medicines [49, 50]. Therefore, ensuring medicine quality within UHC requires planning and continued attention to regulatory processes such as good registration practices and post-market surveillance strategies, as well as robust quality assurance mechanisms. There are also concerns that the costs of medicine quality assurance activities could result in higher medicine prices, making them less affordable. However, other literature has argued that it is feasible to attain UHC with affordable quality medicines through a mix of quality assurance interventions and incentive schemes [51], citing successes such as the WHO prequalification program (WHO PQP) and the Medicines Patent Pool in increasing access to affordable, quality-assured medicines [52, 53]. Our study builds upon this prior literature by illustrating the multiple conceptual linkages and connections between medicines quality assurance systems and UHC processes. Further, our study shows how quality-assured medicines can improve health outcomes and reduce the financial costs of implementing UHC by preventing costs associated with utilization of poor-quality medicines. Hence, evaluations of the potential costs of quality assurance systems in UHC schemes, including possible higher medicine costs, should include the value of the averted health and economic costs of substandard and falsified medicines that would ensue if there are no investments in quality assurance systems. Accrued savings in the health system from utilizing quality-assured medicines could then be reinvested in strengthening various UHC dimensions, including reducing out-of-pocket expenses for patients and making medicines more affordable.

Our analysis has a number of limitations. Agent-based models provide results that depend on the quality of data inputs. Because data on substandard and falsified medicines, care-seeking, and costs for malaria in LMICs are limited, we performed extensive literature searches and analysis of the most recent quality data for our inputs. Epidemiological data and cost inputs were probabilistically ranged to account for uncertainty in outcomes [26]. In addition, our data analysis was limited by data availability across countries where combined indicators were only available for 51 to 63 countries. Given this small number of data points, many confounders could not be controlled for, including country differences in underlying wealth, wealth distribution, population composition, distribution of health services, and political systems. Although the prevalence of substandard and falsified medicines was searched systematically, the prevalence we report were based on a limited number of medicine quality studies performed in each country. In addition, our literature analysis does not include data generated by medicines regulatory authorities through their own post-marketing surveillance processes, which are not readily made publically available.

Despite these limitations, this study contributes to emerging analyses regarding the role that medicines quality assurance systems play in UHC, and conceptually highlights the importance of ensuring medicine quality in order to achieve UHC goals. Our systems map can be used as a conceptual and advocacy tool to make a case for the importance of investing in medicine quality assurance with UHC among a broad range of stakeholders. The system mapping approach can also be adapted and customized to explore medicine quality assurance and UHC within local specific contexts. Cost-savings implications of ensuring medicine quality and providing UHC can assist policy makers, governments, civil society, and other stakeholders, when prioritizing health interventions and engaging with health insurers and other financing agencies. In addition, this analysis highlights the critical role that Medicines Regulatory Authorities play in UHC through several key functions, such as enforcement of good practices for manufacturing, registration, distribution and storage; establishing pharmacovigilance and medicines quality post-marketing surveillance programs; and enforcing regulatory actions to withdraw poor-quality medicines from circulation. Thus, ensuring the use of quality-assured medicines is critical in UHC and investing in medicine quality assurance systems and interventions is vital for the long-term success of UHC planning in LMICs.

## Supporting information

S1 Fig SAFARI model flow diagram. (TIF)

S1 Table Indicators used in data analysis. (DOCX)
