# Supplementary figures and images for: Importance of medicine quality in achieving universal health coverage
Source: PLoS One. 2020 Jul 9;15(7):e0232966. doi: 10.1371/journal.pone.0232966 (PMC7347121; doi:10.1371/journal.pone.0232966)

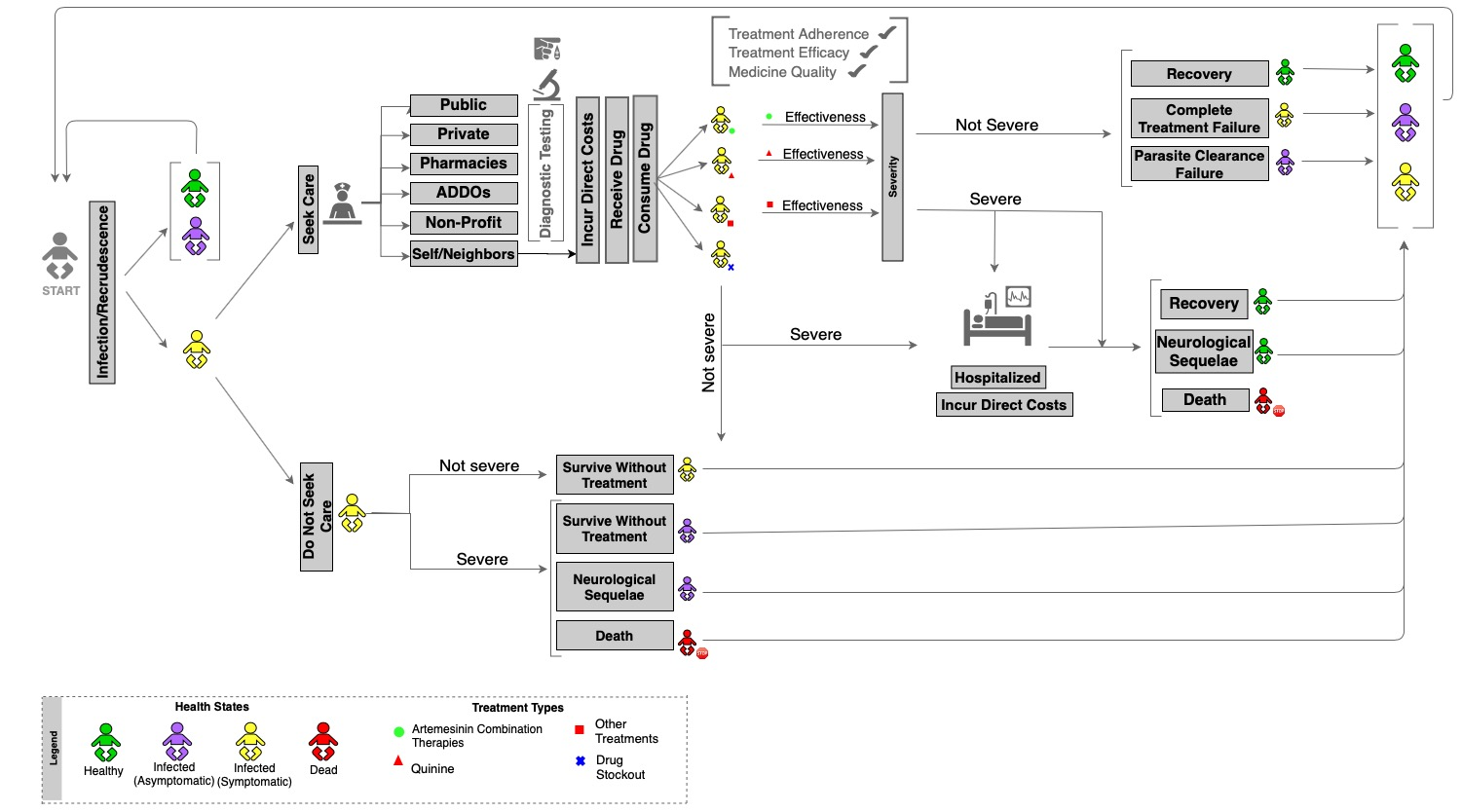

Supplement: S1 Fig — (TIF) [file pone.0232966.s001.tif]
