# Supplementary material for: Importance of medicine quality in achieving universal health coverage
Source: PLoS One. 2020 Jul 9;15(7):e0232966. doi: 10.1371/journal.pone.0232966 (PMC7347121; doi:10.1371/journal.pone.0232966)
Supplement: S1 Table — (DOCX) [file pone.0232966.s002.docx]

**Importance of medicine quality in achieving universal health coverage**

S1 Table. Indicators Used in Data Analysis

|  | **Prevalence of SF Medicines (%) [**[**1**](#_ENREF_1)**]** | **U5MR (per 1000 live births)** **[**[**2**](#_ENREF_2)**]** | **Regulatory Quality Score** **[**[**3**](#_ENREF_3)**]** | **Government Effectiveness Score** **[**[**3**](#_ENREF_3)**]** | **Catastrophic Spending at 10% of HH expenditures [**[**4**](#_ENREF_4)**]** | **Essential Services**  **Coverage (%) [**[**5**](#_ENREF_5)**]** | **GDP Per Capita (US$) [**[**6**](#_ENREF_6)**]** |
| --- | --- | --- | --- | --- | --- | --- | --- |
| **Afghanistan** | 0.1411 | 67.9 | -1.3392 | -1.3256 | 4.84 | 34 | 550.07 |
| **Angola** | 0.2588 | 81.1 | -1.0442 | -1.0288 | 12.38 | 36 | 4100.29 |
| **Armenia** | 0.0952 | 12.6 | 0.2805 | -0.0966 | 16.05 | 67 | 3936.80 |
| **Azerbaijan** | 0.0968 | 23.0 | -0.2457 | -0.1615 | 8.12 | 64 | 4131.62 |
| **Bangladesh** | 0.3019 | 32.4 | -0.8073 | -0.7379 | 13.57 | 46 | 1516.51 |
| **Belarus** | 0.0667 | 3.7 | -0.7362 | -0.3464 | 4.38 | 74 | 5727.51 |
| **Benin** | 0.2062 | 98.3 | -0.4722 | -0.6435 | 11.11 | 41 | 827.39 |
| **Bolivia** | 0.1895 | 34.9 | -0.8987 | -0.3906 | 8.23 | 60 | 3393.96 |
| **Brazil** | 0.0732 | 14.8 | -0.1144 | -0.2879 | 25.56 | 77 | 9821.41 |
| **Burkina Faso** | 0.2174 | 81.2 | -0.4435 | -0.5888 | 3.52 | 39 | 642.04 |
| **Cambodia** | 0.2017 | 29.2 | -0.5029 | -0.6553 |  | 55 | 1384.42 |
| **Cameroon** | 0.2355 | 84.0 | -0.8228 | -0.8198 | 10.78 | 44 | 1451.87 |
| **Chad** | 0.3298 | 123.2 | -1.2102 | -1.4610 | 6.28 | 29 | 662.50 |
| **China** | 0.0455 | 9.3 | -0.1476 | 0.4219 | 17.71 | 76 | 8826.99 |
| **Colombia** | 0.0826 | 14.7 | 0.3409 | -0.0673 | 16.92 | 76 | 6408.92 |
| **Democratic Republic of the Congo** | 0.1441 | 91.1 | -1.4656 | -1.6342 | 5.81 | 40 | 462.78 |
| **Ecuador** | 0.1651 | 14.5 | -1.0023 | -0.3207 | 15.23 | 75 | 6273.49 |
| **Equatorial Guinea** | 0.0901 | 89.6 | -1.3950 | -1.4375 |  | 45 | 9697.63 |
| **Ethiopia** | 0.2749 | 58.5 | -1.0061 | -0.6992 | 0.82 | 39 | 767.56 |
| **Gabon** | 0.0450 | 48.3 | -0.7867 | -0.9432 | 5.67 | 52 | 7413.80 |
| **Ghana** | 0.4091 | 49.3 | -0.1376 | -0.1120 | 3.11 | 45 | 2046.11 |
| **Guinea** | 0.1228 | 85.7 | -0.8379 | -1.0442 | 6.97 | 35 | 823.49 |
| **Guinea-Bissau** | 0.3333 | 84.2 | -1.1819 | -1.7661 |  | 39 | 723.61 |
| **Guyana** | 0.1803 | 31.3 | -0.3934 | -0.2906 |  | 68 | 4655.14 |
| **India** | 0.0513 | 39.4 | -0.2540 | 0.0917 | 17.33 | 56 | 1942.10 |
| **Indonesia** | 0.1827 | 25.4 | -0.1063 | 0.0408 | 3.61 | 49 | 3846.86 |
| **Ivory Coast** | 0.1763 | 88.8 | -0.3619 | -0.7415 | 15.19 | 44 | 1537.50 |
| **Kazakhstan** | 0.1937 | 10.0 | 0.1704 | 0.0089 | 1.83 | 71 | 9030.38 |
| **Kenya** | 0.0959 | 45.6 | -0.2326 | -0.3120 | 5.83 | 57 | 1594.84 |
| **Lao People's Democratic Republic** | 0.2789 | 63.4 | -0.7215 | -0.3587 | 2.98 | 48 | 2457.38 |
| **Madagascar** | 0.2500 | 44.2 | -0.6940 | -1.1377 | 0.77 | 30 | 449.72 |
| **Malawi** | 0.6221 | 55.4 | -0.7485 | -0.6695 | 1.64 | 44 | 338.48 |
| **Mali** | 0.4063 | 106.0 | -0.5727 | -0.9376 | 3.38 | 32 | 827.01 |
| **Mauritania** | 0.1453 | 79.0 | -0.7760 | -0.7202 | 10.54 | 33 | 1136.77 |
| **Mongolia** | 0.1464 | 17.2 | -0.1172 | -0.2619 | 2.39 | 63 | 3717.47 |
| **Mozambique** | 0.1489 | 72.4 | -0.7296 | -0.8927 | 1.19 | 42 | 426.22 |
| **Myanmar** | 0.1215 | 48.6 | -0.8317 | -1.0513 |  | 60 | 1256.66 |
| **Namibia** | 0.1391 | 44.2 | -0.1896 | 0.1987 |  | 59 | 5230.77 |
| **Nepal** | 0.2000 | 33.7 | -0.7160 | -0.8818 | 27.41 | 46 | 849.01 |
| **Niger** | 0.3448 | 84.5 | -0.6793 | -0.6733 | 4.14 | 33 | 378.06 |
| **Nigeria** | 0.1415 | 100.2 | -0.8855 | -0.9604 | 24.77 | 39 | 1968.43 |
| **Pakistan** | 0.2348 | 74.9 | -0.5933 | -0.5850 | 1.03 | 40 | 1547.85 |
| **Papua New Guinea** | 0.1000 | 53.4 | -0.6534 | -0.6578 |  | 41 | 2488.90 |
| **Republic of Congo** | 0.2157 | 47.5 | -1.3298 | -1.1943 | 1.97 | 38 | 1654.01 |
| **Russia** | 0.0364 | 7.6 | -0.4753 | -0.0776 | 4.87 | 63 | 10743.10 |
| **Rwanda** | 0.2443 | 37.9 | 0.1463 | 0.2609 | 4.61 | 53 | 748.29 |
| **Senegal** | 0.1458 | 45.4 | -0.1475 | -0.3198 | 3.33 | 41 | 1329.30 |
| **South Africa** | 0.0889 | 37.1 | 0.2331 | 0.2785 | 1.41 | 67 | 6151.08 |
| **Sudan** | 0.1136 | 63.2 | -1.5604 | -1.4149 |  | 43 | 2898.55 |
| **Suriname** | 0.6410 | 19.6 | -0.5375 | -0.6167 |  | 68 | 5317.39 |
| **Tajikistan** | 0.1600 | 33.6 | -1.0602 | -1.1205 | 11.30 | 65 | 801.05 |
| **Tanzania** | 0.0593 | 54.0 | -0.5793 | -0.6294 | 9.87 | 39 | 936.33 |
| **Thailand** | 0.0945 | 9.5 | 0.1408 | 0.3846 | 3.38 | 75 | 6595.00 |
| **Togo** | 0.3609 | 72.9 | -0.7899 | -1.1253 | 10.65 | 42 | 610.15 |
| **Turkey** | 0.0000 | 11.6 | 0.0351 | 0.0705 | 3.10 | 71 | 10546.15 |
| **Uganda** | 0.2297 | 49.0 | -0.2225 | -0.5842 | 12.01 | 44 | 606.47 |
| **Ukraine** | 0.0377 | 8.8 | -0.3213 | -0.4598 | 7.21 | 63 | 2639.82 |
| **Uzbekistan** | 0.1333 | 22.5 | -1.2606 | -0.5560 |  | 72 | 1533.85 |
| **Venezuela** | 0.4348 | 30.9 | -1.9591 | -1.3990 |  | 73 | 15692.41 |
| **Viet Nam** | 0.4936 | 20.9 | -0.3983 | 0.0024 | 9.81 | 73 | 2342.24 |
| **Yemen** | 0.2800 | 55.4 | -1.4501 | -1.9199 | 17.06 | 39 | 1106.80 |
| **Zambia** | 0.1026 | 60.0 | -0.4661 | -0.6220 | 0.29 | 56 | 1513.28 |
| **Zimbabwe** | 0.1633 | 50.3 | -1.5633 | -1.1906 |  | 55 | 1333.40 |

GDP = Gross Domestic Product; HH = household; SF = substandard and falsified; U5MR = under five mortality rate

References

1. Ozawa S, Evans DR, Bessias S, Haynie DG, Yemeke TT, Laing SK, et al. Prevalence and Estimated Economic Burden of Substandard and Falsified Medicines in Low- and Middle-Income Countries: A Systematic Review and Meta-analysis. JAMA network open. 2018;1(4):e181662. doi: 10.1001/jamanetworkopen.2018.1662. PubMed PMID: 30646106; PubMed Central PMCID: PMC6324280.

2. UNICEF. Child Mortality Estimates New York: UNICEF; 2019 [cited 2019 January 15]. Available from: https://data.unicef.org/topic/child-survival/under-five-mortality/.

3. World Bank. World Governance Indicators. Washington, DC: The World Bank; 2018.

4. UHC2030. UHC indicators for SDG monitoring framework agreed 2017 [cited 2019 March 1]. Available from: https://[www.uhc2030.org/news-events/uhc2030-news/uhc-indicators-for-sdg-monitoring-framework-agreed-398330/](http://www.uhc2030.org/news-events/uhc2030-news/uhc-indicators-for-sdg-monitoring-framework-agreed-398330/).

5. World Health Organization. UHC service coverage Geneva, Switzerland: World Health Organization; 2015.

6. World Bank. GDP per capita (current US$). Washington, DC: The World Bank; 2017.
